# Recognizing the Known Unknowns; the Interaction Between Reflective Thinking and Optimism for Uncertainty Among Software Developer’s Security Perceptions

**DOI:** 10.1037/tmb0000122

**Published:** 2023-11-24

**Authors:** Matthew Ivory, John Towse, Miriam Sturdee, Mark Levine, Bashar Nuseibeh

**Affiliations:** 1 Department of Psychology, Lancaster University; 2 School of Computer Science, St. Andrews University; 3 Department of Computer Science and Information, LERO (Irish Software Research Centre), Limerick, Ireland; 4 School of Computing and Communications, Open University

**Keywords:** software engineer, cognitive reflection, optimism bias

## Abstract

Software development is a complex process requiring aspects of social, cognitive, and technical skills. Software engineers face high levels of uncertainty and risk during functional and security decision making. This preregistered study investigates behavioral measures of cognitive reflection, risk aversion, and optimism bias among professional freelance software developers and computer science students, to expose relationships between uncertainty-associated language and risk sensitivity. We employ content analysis with a mixed-effect model to understand how psychological dimensions influence risk sensitivity in secure software development. We show an interaction between cognitive reflection and optimism bias in the proportion of uncertainty-related language used. Overly optimistic outlooks combined with higher cognitive reflection drives up expressions of uncertainty, while pessimistic or realistic individuals reduce uncertainty as cognitive reflection increases. Software engineers who hold average or pessimistic views on the security of their code are more likely to speak more intuitively about security and risk. We discuss the potential of our findings in relation to understanding how to leverage language used by engineers as markers of risk aversion. Encouraging increased discourse could be used as a catalyst for increased cognitive reflection and grounding optimistic behaviors, leading to more careful decisions.

Software is at the foundation of our modern world; we rely on it for almost everything, from communication to financial transactions, to information storage. When software fails or is altered by malicious actors, this has real-world consequences, including psychological harm (Palassis et al., 2021). Consequently, reducing software vulnerabilities—for example, by applying psychological insights and methods to those who create software—is becoming increasingly important.

Secure coding is defined as a programming practice that avoids software vulnerabilities (Rauf et al., 2021), allowing for data exchanges and logic flows to occur without interference by third parties. For our purposes, a security vulnerability is defined as an unexpected logic flow resulting in exploitable situations allowing for unintended access to information or functionality. Reducing vulnerabilities and increasing risk sensitivity in software engineers therefore has the potential for improving secure coding practices.

The solution appears simple—“Write secure software!,” yet such imperatives are usually not sufficient for secure coding (Hallett et al., 2021). It is well established that warnings or commands alone do not motivate action unless an individual perceives the importance of the command (Dash & Gladwin, 2007). Despite software security’s importance, 76% of software possessed at least one well-known vulnerability in a recent review (Veracode, 2020), and 69% of software engineers were unaware of third-party vulnerabilities within their own code (Kula et al., 2018). Our approach works toward a better understanding of the psychological profiles of those who write software, to ultimately facilitate an increase in the salience of security and deployment of secure practices.

Secure coding can be achieved through technical interventions, such as using dedicated testing teams or rigorous workflows, but with over 40% of the app software engineer community comprising of solo or amateur software engineers (van der Linden et al., 2020), it is important to address the individual within the development process, rather than just roles and technical solutions.

When investigating software engineers’ priorities, functionality has repeatedly superseded security (Kirlappos et al., 2013; Lopez, Sharp, et al., 2019), despite implicit security expectations in high-quality software (Tahaei & Vaniea, 2019). Even when given security-specific coding tasks (such as password storage), security is often omitted unless prompted (Hallett et al., 2021; Naiakshina et al., 2019). A reduced security focus has been attributed variously to a perception of effort (Kirlappos et al., 2013), absence of responsibility (Gotterbarn, 2001), and even to mistaken assumptions that their tools are secure (Nadi et al., 2016; Palombo et al., 2020). To our knowledge, the disposition toward risk and uncertainty has not been systematically explored.

To that end, we use a novel approach to highlight links between domain-general psychological constructs such as cognitive reflection, risk aversion, and optimism biases as mediators of how engineers might think about security. This research emphasizes the need to consider individual differences in the psychology of engineers as their actions can have significant impact within the real world, consistent with the suggestion that heuristics and biases impact secure software decision making (Brun et al., 2022; Oliveira et al., 2014, 2018).

By deliberately using abstract cognitive measures as opposed to signals from software-specific activities, we aim to develop an understanding of how cognition shapes or molds those activities. Through a quantitative analysis of language use around security perceptions, we can highlight links between software security and the language used. Language is grounded in perception and action and can be used to share our internalized models with others (Hagoort, 2023; Jackendoff, 2009). Through studying how software engineers describe security within their work and their personal experiences (or absence of), we can associate their language with cognition, which reflects their internal models and beliefs, and may reflect their real-world behavior, but importantly, it may provide an opportunity to identify potentially insecure coding practices.

## Cognitive Processing Styles

Dual processing theory frames decision making and reasoning in terms of individual propensity to engage in different cognitive styles (Evans, 2003). System 1 processing is driven by intuition and heuristic use, allowing individuals to reduce complex decisions into simpler operations requiring less cognitive effort (Kahneman et al., 1974; Kahneman & Frederick, 2002). Heuristics form “mental shortcuts” that can be used in multiple scenarios to simplify and speedup decisions (Beike & Sherman, 1994). Through heuristic use, System 1 processing is much faster, automatic, and intuitive compared to more drawn-out processing, or System 2. System 2 processing involves deliberate, thought-out, and analytic judgements, allowing for abstract and hypothetical thinking. System 2 is more computationally demanding than System 1, attempting to arrive at optimal solutions by analyzing available information, and is reserved for situations that cannot be resolved with System 1 processing.

A default-interventionist model of dual processing (Evans & Stanovich, 2013; Kahneman & Frederick, 2002) suggests decision making uses System 1 as a default, and System 2 is deployed only when it is sufficiently cued and available (Damnjanović et al., 2019; Evans, 2010b). Both systems can possess conscious and unconscious aspects of cognition (Evans, 2010a).

If System 2 is not sufficiently cued, then decision making relies on heuristics. Gigerenzer (2002) suggested that these heuristics form part of an adaptive toolbox, allowing for rational decisions within the constraints of the available information (bounded rationality). Importantly, a core aspect of the adaptive toolbox is that heuristics can be considered ecologically rational if they benefit decision making within specific contexts (Gigerenzer, 2015). When heuristics fail to provide good choices, it is not strictly the cognitive mechanism itself, but rather its poor fit to the decision context (Gigerenzer & Todd, 1999; Kahneman et al., 1974). This is relevant to writing secure code, as it has been suggested previously that heuristics do not provide rational decisions in these contexts resulting in biased decision making (Oliveira et al., 2018).

Biases can be defined as systematic, flawed response patterns that deviate from expected normative performance (Evans, 1984). Not all heuristics invoke biases and poor decisions, but in certain instances, they can result in nonoptimal decisions. One example of a context where heuristics become biases is software security. In daily life, we possess no security heuristic, and by extension, no heuristic exists for secure coding (Oliveira et al., 2014). Oliveira et al. primed developers for security which increased their sensitivity to software vulnerabilities, dual-processing theory would frame this as priming that triggers System 2 processing. Previous work following the claim that software security is heavily impacted by biased thinking has examined links between general cognition and secure coding (Brun et al., 2022; Oliveira et al., 2018), whereas we build upon this claim by using widely deployed measures of thinking and dual processing to examine the individual differences.

Previous research suggests security prompting can increase code quality (Hallett et al., 2021), specific vulnerability identification (Spadini et al., 2020), and code comprehension (Danilova et al., 2021). Moreover, System 1 processing can be suppressed in favor of System 2 through prompting, either overtly through verbal/written requests (Evans & Stanovich, 2013; Pennycook et al., 2020), or implicitly through metacognitive prompts (Alter et al., 2007; Alter & Oppenheimer, 2008).

So how can we measure the disposition toward types of thinking and biases? We review evidence that *cognitive reflection* examines the activation of the processing systems, *prospect theory* measures risk seeking behaviors, and *optimism bias* is a proxy for intuitive processing.

### Cognitive Reflection

Cognitive reflection is the ability to reflect upon a question before answering and inhibiting the immediate response and is most commonly measured using a form of the Cognitive Reflection Test (CRT; Frederick, 2005). In this article, we take the position that CRT is a useful measure of an individual’s propensity to activate System 2 processing in response to a question requiring more deliberate thought, and by virtue of engaging system 2, is also a measure of System 1 suppression (Frederick, 2005).

Specifically, the CRT has been widely deployed as a measure of System 2 engagement, through the suppression of System 1. The CRT presents questions with intuitive, yet incorrect answers and one must reflect on the question to respond correctly. An example of a CRT question is, “A bat and a ball cost $1.10 in total. The bat costs $1.00 more than the ball. How much does the ball cost?”^1^ The intuitive and incorrect response is the most common answer (Frederick, 2005; Sinayev & Peters, 2015), implying that indeed System 1 thinking is a default mechanism.

CRT has been informative for illuminating decision making in the workplace. Teachers in higher education demonstrated higher CRT being associated with teaching more technology-related materials (Janssen et al., 2019), implying educators with higher reflective thinking are more suited to teaching fields that require high levels of logical thinking (such as information technology). In terms of teaching experience, CRT was associated with increased rationality and greater consideration of future events (Čavojová & Jurkovič, 2017).

To our knowledge, there is no empirical work investigating CRT and software development, however, CRT has been linked with predicting susceptibility to detecting phishing emails (Jones et al., 2019), loss aversion (Frederick, 2005), and forecasting (Moritz et al., 2014).

### Optimism Bias

Bias susceptibility can be used as a proxy to capture the strength of System 1 processing. Unrealistic optimism is a bias where individuals perceive themselves to be less likely to experience negative events compared to others (Sharot, 2011; Weinstein, 1980). People typically exhibit a persistent bias regarding future events, in that they overestimate the likelihood they will experience positive events and underestimate the chances of experiencing negative ones. This bias covers a broad range of events, from life events such as car accidents to more mundane events, such as estimating the time required to complete a project (Cappos et al., 2014).

For cybersecurity, users have been found to underestimate their risk online, increasing their vulnerability cybersecurity attacks (West, 2008; Wiederhold, 2014). In a thematic analysis of the same data presented in this article, engineers reported negative events as being damaging to their identity as an engineer (Ivory et al., 2023), indicating that for some, they associate the potential of software vulnerabilities in one’s code as being a negative event. As such, a logical assumption would be that software developers are also susceptible to optimistic outlooks toward their work, and underestimating the likelihood of negative events, such as vulnerabilities in their code (as a potential marker of low-quality work).

Optimism in the software development domain has been previously investigated using professionals, with software engineers being worse at estimating time management than those in nontechnical roles (Mølokken & Jørgensen, 2005). Both software engineers and executives experience difficulties in making security-related decisions, demonstrating overconfidence in their software security (Loske et al., 2013). We extend the understanding of optimism bias in a security-specific context by relating this to the language used around software security.

### Risk Aversion

Risk aversion can be linked to prospect theory, which suggests decisions maximize gains and minimize losses (Kahneman & Tversky, 1979). People experience negativity from a loss more strongly than positivity from gains. Accordingly, people typically make choices that minimize loss over maximizing gain (Levy, 1992).

Frederick (2005) reported an association with prospect theory and CRT, showing higher CRT scores accompanied increased risk aversion to losses (i.e., an increased willingness to accept a sure loss than to risk a greater loss). Frederick also showed people make riskier decisions when facing potential gains (willing to risk greater gains than accept sure gains). This supports the idea risk aversion is influenced by heuristics and cognitive biases. Risk aversion has wider generalizability toward, for example, general decision making under risk (Abdellaoui et al., 2007).

Kina et al. (2016) examined software engineer tool adoption practices and found that when new tools are not guaranteed to maximize profit and contain risks, engineers are more risk averse and instead rely upon the known familiarity of familiar tools. This suggests a certainty effect, or bias in play. When examining factors that shape software project decisions, loss magnitude was considered a more significant factor than loss likelihood, highlighting the relevance of choice value (Keil et al., 2000). By measuring risk aversion as a function of risk sensitivity in language used when talking about security, we can examine how security is framed and how the framing manifests in the language used.

## Motivations

There have been calls for more psychological research within computing and software security (Acar et al., 2016; Capretz & Ahmed, 2018), with greater appreciation of the individual involved, and the heterogeneity of behaviors relevant to development roles. The present research answers these calls by exploring the role of individual cognition and its effect on security perceptions. We address this by investigating the cognitive differences seen in risk sensitivity when talking about security in software. Within software engineering, the role of biases and heuristics has been acknowledged (Chattopadhyay et al., 2020; Petre, 2022), as well as in a systematic review, while noting that research has yet to fully characterize and describe their impact (Mohanani et al., 2020). Psychology is well positioned to respond to this gap through empirical research. Through cognitive measures and the analysis of individual differences, we can apply dual processing theory to better understand how this ultimately explains perceptions of security within software development.

Two different populations were included in our sample, freelance developers, and computer science students, as they represent two different timepoints in the software engineer lifecycle. Students represent early stage, less experienced engineers, and freelancers represent those who have experience from prior projects. The motivation for using both populations is to better understand how security perceptions and risk sensitivity may change (or not change) over the lifecycle of engineers. Increasing awareness of the cognitive features that underpin security perceptions in software across the engineer lifecycle, we are better placed to provide interventions at the most effective points. If risk sensitivity is similar across both populations, then intervening as early as possible may be the solution, whereas if a difference is seen, indicating an event or events altering risk sensitivity, then interventions should look to address these events.

This article is part of a larger project (Ivory, 2022) that examines both cognitive links and risk perception, alongside social identity, and responsibility within software development. The project comprises two independent, self-contained components, with the complementary package focusing on a thematic analysis of responsibility and risk acceptance (Ivory et al., 2023). The overall project information and data can be found at https://doi.org/10.17605/OSF.IO/P6DY5.

### Hypotheses

Three hypotheses were created to direct this research:

*Hypothesis 1:* Higher risk aversion scores will be associated with increased awareness and sensitivity to risk in language describing professional work related to software development. Lower risk aversion (higher risk attraction) will be associated with less awareness and sensitivity to risk in language describing professional work.

*Hypothesis 2:* Higher cognitive reflection test scores will be associated with increased awareness and sensitivity to risk in language describing professional work. Lower cognitive reflection (higher risk attraction) will be associated with less awareness and sensitivity to risk in language describing professional work.

*Hypothesis 3:* Mean scores closer to zero on the novel OWASP^2^ risk task will be found with higher scores of cognitive reflection. Scores of zero are expected to represent a realistic, unclouded view of risk which should align with more reflective thinking styles.

## Methodology

### Participants

As per the preregistration, we required a minimum sample of 122 participants, 61 from each population to meet a desired power level of 80%. A sample of 150 were sought to account for low-quality responses. We recruited 149 participants and excluded data from eight participants; four failed over 50% of attention checks, one preferred not to provide gender information (one observation would not be appropriate to use in analysis to make meaningful implications), and three provided responses of less than 20 words to the questions. Our data corpus thus comprises 141 participants (“software engineers”), 69 of whom were professional freelance software (six females and 63 males) developers and 72 were computer science (CS) students (31 female and 41 male).

All research involved in this project was approved by the University Faculty Ethics committee. Participants provided informed consent before commencing with the research study.

We recruited developers using the freelance website, https://www.upwork.com/. We uploaded an advertisement (available on OSF; https://doi.org/10.17605/OSF.IO/P6DY5) asking participants to complete a study about their understanding of security in software development. We specified that participants should be currently working in software development, have experience writing secure code and have been involved in noneducation-based software projects. We compensated freelance developers at rate of £10/hr. The student sample was collected from two sources, using internal university mailing lists and the recruitment website, Prolific. We compensated CS students at a rate of £8.50/hr.

**Figure 1 fig1:**
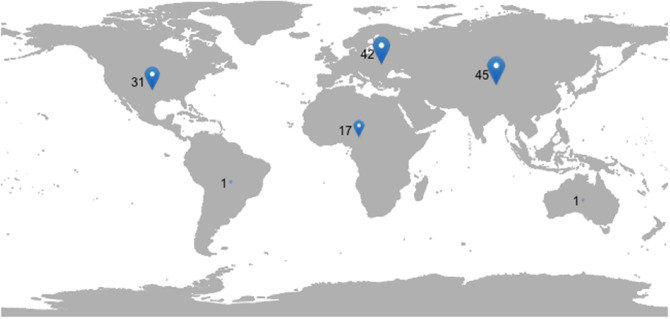
Map Displaying Approximated Location/Nationality Data of Participants on a Continent Level *Note*. This approximates location and nationality of each participant due to expected immigration movement. “Participant Distribution” by Matthew Ivory, licensed under CC BY 4.0 from https://doi.org/10.17605/OSF.IO/P6DY5.

Age details can be found in Table 1. Due to data collection issues, nationality data for developers are unavailable, which was substituted with country-level location. Figure 1 shows an approximation of participant location and nationality by presenting continental-level data. Most people do not emigrate from their origin country, with around one in 30 migrating internationally (McAuliffe & Triandafyllidou, 2021). Representing both nationality and location together approximates both data types on a continental scale. Ethnicity and socioeconomic status were not collected in the study design and cannot be reported or discussed.[Table tbl1][Fig fig1]

### Materials

Participants completed the study through the online surveying software Qualtrics. The survey (in study presentation order) included demographic information, an Open Web Application Security Project (OWASP) vulnerability task (OVT), four open-text response questions focused on risk awareness, aversion, and mitigation in software development, a gambling task and two versions of CRTs (Frederick, 2005; Thomson & Oppenheimer, 2016).

#### OWASP Vulnerability Task

The OVT is designed to measure unrealistic optimism in software security by leveraging familiar concepts, such as well-known vulnerabilities that are well established and consistently highlighted year-on-year. It can be expected that software engineers will perceive including vulnerabilities in their own code as a more negative event than vulnerabilities created by other developers. The OVT builds upon the finding that individuals tend to underestimate the likelihood of negative events affecting them (Sharot, 2011; Weinstein, 1980). While previous cybersecurity research has focused on users and their optimism (West, 2008; Wiederhold, 2014), we devised a measure that is specific for software developers who engage in secure coding. The OVT is a measure of comparative optimism between an individual’s estimation of their own likelihood of including security vulnerabilities compared to an average developer.

The measure uses the 2021 top five vulnerabilities: Injection flaws, Broken Authentication, Sensitive Data Exposure, XML External Entity flaws, and Broken Access Control.^3^ The top five vulnerabilities were used as opposed to the full list of 10 as less experienced developers are more likely to be familiar with these vulnerabilities.

The OVT consists of two parts, separated by a secondary task, and presented in a randomized order to reduce recall effects. The first asks participants to estimate the percentage likelihood of a specific vulnerability existing in web applications released by the “average developer.” The second part asks respondents the likelihood of themselves introducing these vulnerabilities into their own code. Similar measures have been used previously, asking participants for two measures of success, with the first being about personal confidence in correctly answering a question, followed by a second asking about the percentage of other people who would answer correctly (De Neys et al., 2013; Frederick, 2005; Hoover & Healy, 2019).

#### Perceptions of Software Security

The qualitative questions consisted of four items asking participants about their experiences, thoughts, and opinions on security within software development. Participants had the option to write answers or record audio responses using Phonic (https://www.phonic.ai). Participants were asked to write for at least 4 min or speak for 2 min per question. The questions asked are shown in Table 2.[Table tbl2]

#### Risk Aversion

The gambling exercise replicated that used by Frederick (2005). Thirteen questions sought preference between two options of gaining or losing money. Eight questions contrasted gain scenarios (“Gain £100 for sure or a 90% chance of £500”), and five contrasted loss scenarios (“Lose £50 for sure or a 10% chance to lose £800”). All questions were presented in a randomized order.

#### Cognitive Reflection

We employed two CRTs. The original CRT (Frederick, 2005), and the CRT-2 (Thomson & Oppenheimer, 2016), an alternate version designed both to reduce the numerical nature of the questions and address floor effects. We reworded question texts, altering names and values to mitigate attempts to answer questions through an online search for a matching string. The core principle of each question was not modified. An example is changing the word “bat” and “ball” for “article clip” and “elastic band,” respectively. We checked whether participants had seen or answered CRT questions before, although previous research (Białek & Pennycook, 2018; Stagnaro et al., 2018) suggests scores are stable across repeat exposure. We recorded response times to the CRT questions and instructed participants to complete the CRT questions as fast as possible to reduce internet searching.

## Procedure

**Figure 2 fig2:**
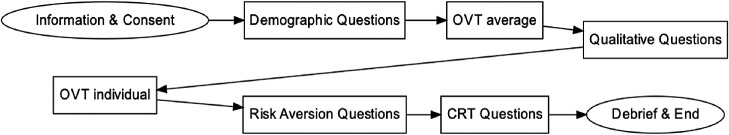
Survey Flow Representing the Presentation Order of Measures Given to Participants *Note*. All participants received the same order of measures with randomization within the measures (detailed in text where appropriate). OVT = Open Web Application Security Project vulnerability task; CRT = Cognitive Reflection Test.

Participants were presented through Qualtrics with an information sheet that described aims and intentions, following which they gave their informed consent. Participants then answered demographic questions, completed the first OVT for the average software engineers, split by the qualitative questions, then completed the second OVT. This was followed by the gambling task and the CRT measures. Finally, participants were presented with a debrief sheet that provided further information, references, and contact details for the researchers. The survey flow is shown in Figure 2.[Fig fig2]

### Research Design

The study comprises a between-participants observational survey design grouped by a two-level factor of population (freelancer vs. student). Data from two independent populations were collected and contrasted. Data validity can be seen in the OSF repository additional online materials.

#### Data Operationalization

Question responses were operationalized through content analysis. As participants had been asked to write for at least 4 min or speak for 2 min per question, we did not expect short responses. The average response wordcount for developers were on average 81.09 words long (*SD* = 61.71), and the students’ responses were longer at 94.38 words (*SD* = 38.38). As a preregistration deviation, we removed responses of 20 word or less (forming a proxy of low quality) as they were less than one standard deviation from the average response (for developers). Of the short responses, 38 were removed in total, from 22 participants. Two participants (both freelancers) had all responses removed, two participants had three responses omitted, six had two responses removed, and 12 participants had only one response removed.

Responses were unnested into single words, words that appeared five or fewer times were removed, remaining words were stemmed to their shortest form (e.g., “secured” and “secure” were stemmed to “secur”). Stopwords (“and,” “because,” “though”) were removed to reduce noise. Then using the Computer Science Academic Vocabulary List (Roesler, 2020), words were tagged as computer science/software relevant. The remaining words were manually tagged for topics of uncertainty, software products, workplace specific language, finance, geographic references, legal, and shallow skills. Following coding, all tagged words were reviewed for appropriateness, and discussed among the research team. Two independent researchers were given a sample of all the retained words and asked to categorize them using the finalized tags, resulting in 20% of all words being reviewed independently (each researcher reviewing 10%).

Interrater reliability was initially assessed through Cohen’s κ (Cohen, 1960). Yet, this measure does not perform well when handling imbalances in the marginal distributions of confusion matrices (Warrens, 2014). In the present case of assessing the reliability of topic coding, the distributions are greatly skewed in favor of negative ratings from researchers, Consequently, small deviations from the positive-positive ratings greatly affect the value of Cohen’s κ. Since percentage of raw agreement is not reliant on marginal distributions (von Eye & von Eye, 2008) we use this as a preferred and complementary measure of interrater reliability.

To calculate κ and percentage agreements, confusion matrices were constructed with the primary researcher’s ratings against the combined ratings of the secondary researchers. As the secondary researchers rated separate sections of the data, there was no overlap between the words and so were combined. From this the percentage of both parties identifying a word belonging to a topic or not provided the interrater reliability. We report the raw percentage agreements in Table 3 as it provides a coarse-grade assessment of potential bias, and we provide the data and the analysis code in the online repository at https://doi.org/10.17605/OSF.IO/SJ8BT (Ivory et al., 2022), providing full transparency of data reliability.[Table tbl3]

Following the reliability assessment, proportional variables were then calculated through topic occurrence divided by word count, creating the variable “proportion of uncertainty-related language” (PURL), as well as proportion of computer science language. This resulted in a value of topic proportion for each question per participant.

#### Data Transformations

We used data transformations to prepare variables for suitable analysis. We scaled continuous variables, CRT, OVT, Gambling scores, PURL, and proportion of computer science language between 0 and 1. We transformed variables with substantially nonnormal distributions to improve normality (see Table 4): OVT scores and PURL scores (with values of zero removed—see the Analytic Strategy section). These transformations are a deviation from our preregistration, and for transparency, additional online materials are available at https://doi.org/10.17605/OSF.IO/SJ8BT that detail the reasons for requiring these transformations. In short, models that used the untransformed data suffered violations of model assumptions and were less effective than models that used the transformed data.[Table tbl4]

### Analysis

Analysis was conducted in R (Version 4.1.0). Data, analysis scripts, and instructions for reproducing the results seen in this article can be found in the OSF repository here at https://doi.org/10.17605/OSF.IO/SJ8BT.

#### Analytic Strategy

To address hypotheses one and two, a two-step model was employed due to the zero-inflated nature of PURL induced through a floor effect (i.e., not all responses used language around uncertainty). The first step was a logistic mixed-effect regression model to determine the existence of uncertainty-related language based on other variables. The second step was a linear mixed-effect regression model to understand the impact of cognition on PURL. For addressing Hypothesis 3, a linear model was built to compare vulnerability scores by cognitive reflection.

Mixed-effect models were used as these handle nested data appropriately. As each participant provided four different text responses, it is important to treat these as independent to each other, but dependent on the participant. This allows for greater model fit, and accounts for participant-level variation in PURL as well as at the question level.

## Results

### Addressing Hypotheses 1 and 2: Individual Differences in Risk and Reflection in Software

To test Hypotheses 1 (that generalizable risk perspectives increase risk sensitivity) and 2 (that generalizable reflective decision-making increases risk sensitivity), a two-step or hurdle model, was developed to first identify the presence of uncertainty-related language, followed by the second model for determining PURL, including risk aversion (as operationalized through gambling task scores), and cognitive reflection (operationalized through CRT scores). Hypothesis 1 is assessed through the inclusion of the loss aversion scores in ensuing models, and Hypothesis 2 is assessed through the inclusion of CRT terms in the models.

In the two-step model, the first model represents a hurdle to be passed, which determines whether uncertainty is reflected in the text. If uncertainty is present and the hurdle is “cleared,” the second model provides predictive power for PURL in the zero-truncated data. Exploratory results that were not preregistered are included in the additional online materials found on OSF.

#### Step 1

The first step was a binary mixed-effect logistic regression model to identify the presence of uncertainty-related language. Not all sentences written by participants included uncertainty, and so PURL distribution was zero-dominated. This model presents the first hurdle, and if the threshold of zero language is crossed, then data are subject to the second step.

This model was developed using a forward-step approach starting with the null model to model the existence of uncertainty language within responses. For model refinement, outliers were identified using Cook’s distance, which measures the influence specific datapoints have over the model fit. Items with a value three times greater than the mean of all the distances were flagged and removed. The final model, with coefficients seen in Table 5, had an Akaike information criterion of 2515.00, explained variance of $R_{\text{conditional}}^{2}$ = .23 and correct classification of 68.13% of data.[Table tbl5]

#### Step 2

**Figure 3 fig3:**
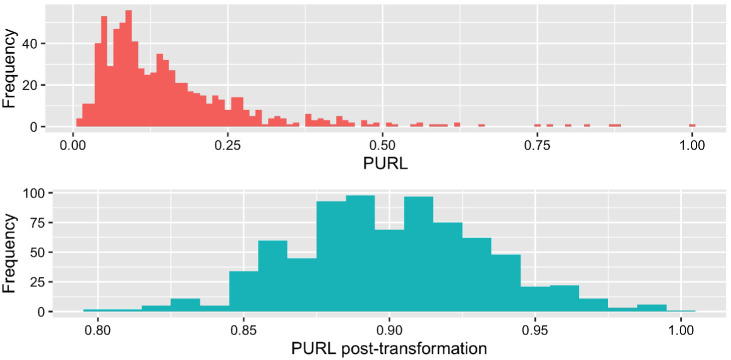
Distribution of the PURL Scores Before and After Transformation Toward a Normal Distribution *Note*. The contrasting plots provide a visualization of the difference in distribution made from the transformations. PURL = proportion of uncertainty-related language.

The second step focused on data containing PURL. This enabled a mixed-effects linear regression model for predicting the amount of PURL in a response. The dependent variable, PURL was zero-truncated, assessed for normality, and transformed toward a more normal distribution. Figure 3 shows the zero-truncated distribution, and the subsequent transformed distribution. The nontransformed zero-truncated data’s Shapiro–Wilks value = .75, *p* < .001, and the transformed data, *W* = .99, *p* = .013. While still nonnormal, it is less right-skewed than the untransformed data. Appendix provides examples of how PURL manifested in responses.[Fig fig3]

Model building began with the null model with terms added sequentially. A mixed-effect model was chosen due to the data’s nested structure. Each independent datapoint indicated a sentence nested within a response, which were nested under participants. For this, a random intercept of participant was included, with a random slope of question to allow for individual differences in language use.

Hat values were used to identify high leverage points, which are datapoints that measure the distance from the observed and fitted values, higher values indicate higher leverage, and we examined values two times greater than the mean hat value. Little relationship was seen, and removal provided negligible effect on the model. Removing influential outliers through Cook’s distance had little effect on the model and so all items were retained.

**Figure 4 fig4:**
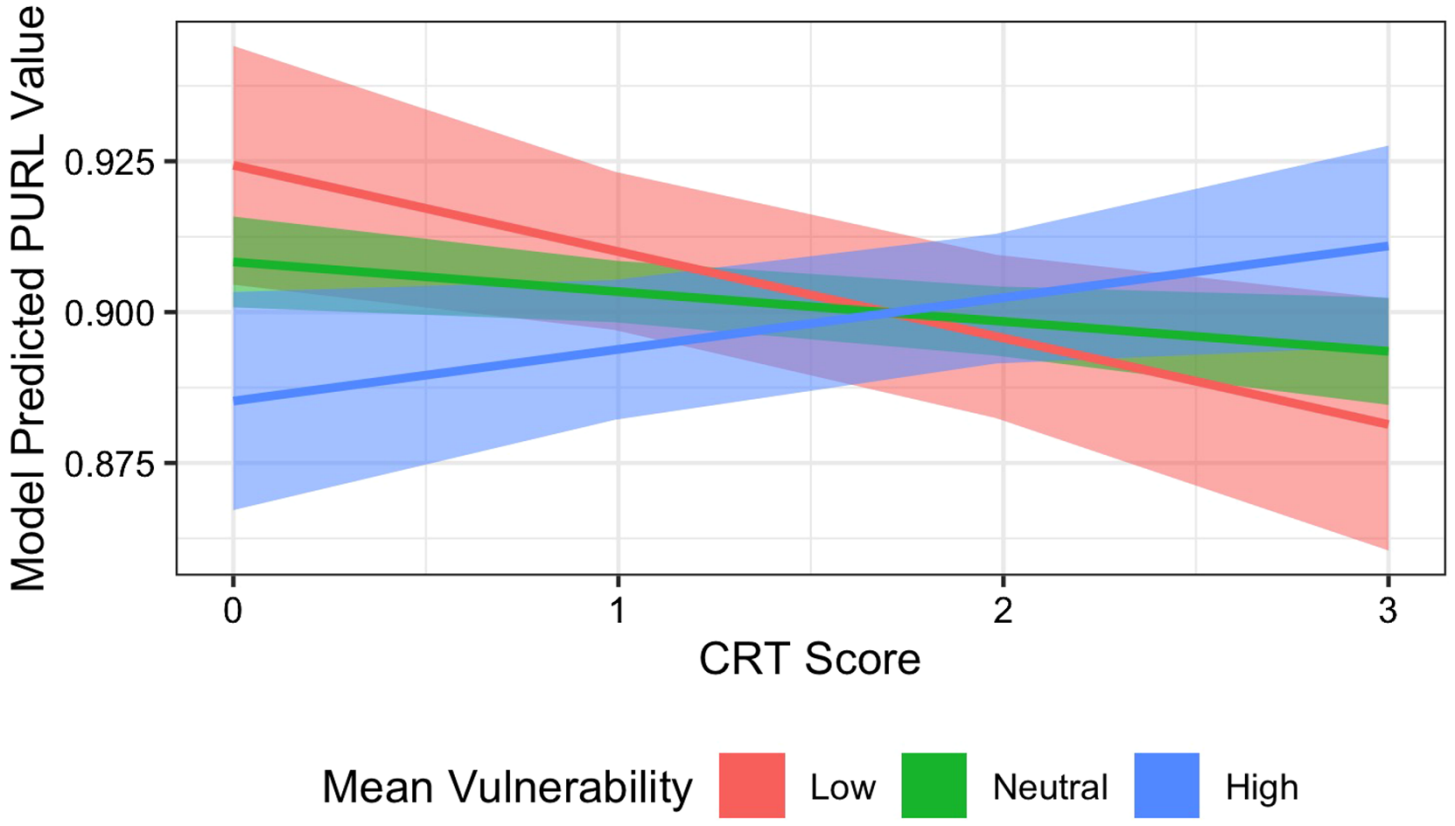
Interaction Plot Between CRT Score (the Propensity for Reflective Thinking) OVT Score (Optimism Bias) and Model Predicted Values *Note*. To provide easier interpretation of the interaction, the plot presents three “groups”—of low, neutral, and high OVT scores. In reality, this grouping does not exist and is a continuum, but the grouping represents the extremes effectively. OVT = Open Web Application Security Project vulnerability task; PURL = proportion of uncertainty-related language; CRT = Cognitive Reflection Test.

The final model can be seen in Table 6 and reports an $R_{\text{conditional}}^{2}$ of .66. The absence of risk aversion scores in the final model indicates no support for Hypothesis 1, and the inclusion of both a CRT term and CRT interaction with optimism supports Hypothesis 2. The model effects are shown in Figure 4, which shows predicted PURL levels for varying strengths of optimism bias. The figure shows a distinct interaction between the optimism bias when measured alongside cognitive reflection. The three levels of OVT presented (low, neutral, and high) serve solely as a visualization aid and not actual groupings made in the analysis. When cognitive reflection was low, PURL was also low, however, as the more optimistic participants increased in their reflection, they spoke more to uncertainty when discussing software. Those displaying an average, or pessimistic view on vulnerability, tended to speak more about uncertainty intuitively, but cognitive reflection suppressed this language, and in extreme pessimism suppressed language to similar levels of the overly optimistic and intuitive.[Table tbl6][Fig fig4]

### Addressing Hypothesis 3

To test for the effects of optimism bias in software development, first a simple linear regression compared OVT scores between students (*M* = 16.72) and developers (*M* = 18.10) which was found to be nonsignificant, *F*(1, 139) = .12, *p* = .727, $R_{\text{adjusted}}^{2}$ = −.01. To test whether software engineers demonstrate a general optimism bias to vulnerability inclusions, a one-sample *t* test was conducted against a true mean of 0. It was found that the mean score calculated across both developers and CS students, 17.40, 95% CI [13.51, 21.28], was significantly different from an average of zero, *t*(140) = 8.86, *p* < .001.

To test Hypothesis 3, a linear regression model was used to predict OVT scores using both CRT measures. A series of models were built to identify the most parsimonious model using Akaike information criterion as the suitability criterion. No model was constructed that provided a significantly better fit than the null model, indicating no strong relationship between OVT scores and cognitive reflection scores. Consequently, we do not reject Hypothesis 3’s null hypothesis that there is no relationship between OVT scores and CRT.

## Discussion

We asked whether domain-general, psychological measures of cognitive reflection, risk aversion, alongside optimism bias for security vulnerabilities would predict how software engineers talk about risk perception when discussing their software security. Using data collected from freelance software developers and CS students, we analyzed PURL through a mixed-effects hurdle model, The key finding was an interaction between cognitive reflection and unrealistic optimism for PURL. Also, engineers were typically overly = optimistic about personal susceptibility toward security vulnerabilities. Meanwhile, risk aversion was not a strong indicator of PURL, nor did we find a systematic relationship between cognitive reflection and optimism.

### Answering the Call for Increased Psychology in Software Engineering Research

This article answers calls for increased psychology-based research in software development (Acar et al., 2016; Capretz & Ahmed, 2018) by showing that software engineers are subject to the same cognitive constraints as nonengineers. The language used by engineers talking about secure coding can be usefully framed with respect to dual-processing theory.

Additionally, a systematic review (Mohanani et al., 2020) within software engineering points toward the breadth of biases affecting software (Fagerholm et al., 2022; Ralph, 2011). Our quantitative, data-driven study extends the understanding of heuristics and biases for secure software. Moreover, the present study underscores the potential for different cognitive constructs to interact in the software domain, revealing the complexity of how heuristics can shape software risk.

### Risk Aversion

Prospect theory proposes that people are more sensitive to losses over gains (Frederick, 2005; Kahneman & Tversky, 1979). As a group, software engineers show this asymmetry too, yet we did not find evidence to support Hypothesis 1 that greater risk aversion would associate with greater risk sensitivity in language. There are two interpretations we present that may explain this finding.

One interpretation is that engineers do not systematically frame security decisions in terms of gains or losses. Software security is often made up of smaller, independent decisions, each with their own framing and outcome value. Typically, software engineers perceive security as a barrier (Lopez et al., 2022), or lower priority than functionality (Lopez, Sharp, et al., 2019), but this may not translate well into mental models of software, meaning that security is poorly considered in terms of risk aversion. It could also be interpreted that risk aversion is unrelated to risk taking behavior as there are other factors that determine the behavior. In economic research, it is seen that low risk aversion can lead to riskier trading decisions (Hoffmann et al., 2015), which contrasts with our expected findings. Further research and a better understanding of the link between software developers’ language and risk behavior is warranted to better understand this finding.

### Cognitive Reflection

The lack of evidence for a direct relationship between CRT and risk sensitivity might reflect (a) the study design, (b) the lack of sensitivity of CRT to capture relevant individual differences, or (c) a more complex relationship than a bivariate link. We believe the evidence points to (c) because we obtained an interaction between CRT and optimism for risk sensitivity. Moreover, this interaction negates the likelihood that (a) or (b) are wholly satisfactory.

The implication is that the specific simple and direct relationship between cognitive reflection and uncertainty language is weak, and a software engineer’s ability to reflect on decisions does not manifest in the language used concerning security. Fine-tuning either—or both—of these constructs might enable stronger evidence to emerge of their link. However, we did find an indirect relationship between CRT cognitive reflection and risk sensitivity in language, one that supports the broad thrust of the initial prediction.

### Optimism

Experienced freelancers and CS students collectively demonstrated a significantly greater optimistic belief in their own secure coding behaviors (see Rhee et al., 2012; Weinstein, 1980) with no noticeable differences between the populations. Measuring unrealistic optimism can serve as a proxy of System 1 processing, as the presence of one bias indicates an increased likelihood of other biases being present too (Ralph, 2011). Insofar as both developers and CS students reported similar levels of optimism, the present research supports the findings of Oliveira et al. (2018) and Brun et al. (2022) that experience does not reduce susceptibility in biases in vulnerability detection.

Observing optimism bias in both new and experienced software engineers reinforces our conclusion that this is an intuitive, instinctive perception. It is neither simply a naïve aspiration nor a survivor bias.

### Interaction: Cognitive Reflection and Optimism

The second step in the hurdle model focused on relationships between cognition and risk sensitivity. The key finding was an interaction between cognitive reflection and optimism that provided predictive power for uncertainty-related language. Those who displayed high optimism spoke less frequently about uncertainty, but as cognitive reflection increased, so did uncertainty. Conversely, pessimistic software engineers frequently mentioned uncertainty, but decreased in uncertainty as cognitive reflection increased. In other words, the way that engineers talk about uncertainty and security is dependent on multiple facets of cognition. This provides some support for Hypothesis 2 but not in its entirety.

The finding that cognitive reflection or optimism alone explain very little variance in security perceptions highlights the entangled nature of cognition in the real-world. Despite cognitive reflection and optimism being two clearly defined measures of cognition within the psychological literature, in data collected from a complex domain, we see that these aspects of cognition are linked and cannot be considered totally modular.

Those naturally optimistic about security, and who deploy System 1 thinking, are less likely to discuss uncertainty—perhaps because it is not seen as an issue. Indeed, Assal and Chiasson (2018) found that overly optimistic software engineers view additional security implementation as holding minimal value, as they believe their current level of security to be sufficient. When considering security, therefore, they may require stronger cues or framing to activate System 2 when making security decisions. Meanwhile, those naturally pessimistic about security, talked more about uncertainty and here System 2 thinking is associated with increased uncertainty (Siegrist et al., 2005).

Reduced self-confidence may mean engineers feel overwhelmed when presented with security decisions, which is not helped by a lack of documentation supporting their choices (Acar et al., 2017) or lone working environments (van der Linden et al., 2020). By activating System 2 processing and thinking critically about security decisions, the naturally pessimistic may find increased confidence in their abilities to code securely. One catalyst for System 2 thinking during secure decision making might be peer communication (Shreeve et al., 2022), which allows for the balancing of perspectives and a reduction of biases. Both examples of the naturally optimistic and pessimistic aligns with the dual-processing theory (Evans, 2003), suggesting that software engineers who can easily activate System 2 processing will experience reduced biased judgment during secure decision making.

A practical ramification of this interaction is that discussions within development teams may help to improve security decisions. While developers working alone do not have access to such support, engagement with interactive websites—such as Stack Overflow—can provide a digital community to engage with (Lopez, Tun, et al., 2019) beyond a simple question-and-answer format. Encouraging developers to interact even asynchronously can potentially provide a community that motivates more reflective thinking. Developing an understanding of how social identity affects these communities and increases engagement between peers can help on this front.

### Rewarding Secure Behaviors

Our findings have implications for how software engineer freelancers (as the focus of this research) are rewarded for practicing secure coding. The implications are also relevant for engineers working in more permanent roles within a company. Rewards can be used as an incentive to encourage certain behaviors and reduce unwanted ones. Rewards can be intrinsic or extrinsic; intrinsic rewards are those internal to the individual and are inherently found in the task itself and upon completion, and extrinsic rewards are external to the task, such as pay or recognition (Ajila & Abiola, 2004).

Intrinsic rewards can boost task performance with motivation also playing a mediating role (Manzoor et al., 2021). Intrinsic rewards can stem from expressions of appreciations by senior employees or promotions based on work quality, and so in nonfreelance engineering roles, this is easily managed by ensuring that employees are recognized for their secure coding practices. Motivation for accuracy or quality can lead to a greater expenditure of cognitive effort (Kunda, 1990), and these motivations can be achieved through an expectation to defend or explain one’s decisions (Tetlock & Kim, 1987).

Extrinsic rewards are not as straightforward for freelance engineers as they can be for contracted engineers, as the companies may be less inclined to provide incentives for secure code if they do not see freelancers as part of the company. One way to provide an extrinsic reward is through the recruitment platforms that they use. If freelancers can be publicly recognized for their secure coding practices in the previous work, this may be seen as a potential reward for quality work as it boosts their profile, increasing potential work offers. The exact nature of verifying secure coding practices on freelance platforms is beyond the scope of this article, but if a system can be devised that is universally trusted and easily implementable, then the use of gamification may work as a reward system. Gamification methods have been shown to have long-term behavioral effects (Hamari, 2017), but gamification should be done with caution as not all findings support their efficacy (Barreto & França, 2021). If badges are perceived by platform-users meaningful and trusted, these may act as a reward system that can be used for freelancers.

The use of rewards, either intrinsic or extrinsic, for encouraging secure coding behaviors can be used to increase motivation and subsequently increase task performance. In a secure coding specific context, by rewarding secure behaviors, this can be linked to greater cognitive engagement, and through the dual-processing theory, lead to reduced bias interference. In the interaction seen between cognitive reflection and optimism, by increasing the likelihood of engaged System 2 processing, clients, stakeholders, and employees can focus on managing optimism rather than juggling two aspects of cognition at a time.

### A Comparison Between Professionals and Students

The finding that population was not a significant term in the modeling process supports the assumptions of our key take-home message, that the data offer credibility for using CS samples in future research in place of professional developers.

The persistence of biases and similar approaches to uncertainty around security implies these are general and widespread characteristics, rather than something unique to students that is modified by experience. This signals that psychological interventions may have an effect across the software engineering domain, and not limited to inexperienced or early stage developers. It also shows that individuals who may be predisposed toward more intuitive, impulsive modes of decision making are not impervious to learning, or seeking support from their work environment to ensure that they make appropriate choices at key points during projects.

Acknowledging that professional developer populations can be harder to access, easier access to a population whose only significant difference is experience provides momentum to further research projects. CS students provide an alternative, cheaper, and easier way to explore hypotheses before confirming findings in professional developers.

Insofar as we proposed novel quantitative hypotheses that addressed general psychological constructs, we have no specific reason to expect that participant diversity (e.g., with respect to the age, gender, and location) would affect performance. Moreover, the profile of these variables broadly corresponds with those of the community—most of our participants were aged 18 and 35 for both groups, which aligns with the Stack Overflow 2022 (https://survey.stackoverflow.co/2022) results that reported over 63% of respondents were aged between 18 and 34. Similarly, the gender split reported by our sample was 74% male compared to Stack Overflow’s reported 92% male audience. This indicates either a potential shift in the future gender split of software developers, with more female developers currently learning to develop software, or it reflects a potential gender split in survey response propensity with more females choosing to participate in nonstandard freelance work offers. The sample was globally distributed representing the global, diverse nature of software development.

### Reflecting on Heuristics

Our research is built upon the theoretical foundations of dual-processing theory and heuristics. We define heuristics as “mental shortcuts” that can be used in multiple scenarios to simplify and speedup decisions (Beike & Sherman, 1994), but when heuristics do not provide appropriate decisions for certain contexts, then they become biases (Gigerenzer, 2015; Kahneman et al., 1974).

Two main schools of heuristics exist, both of which share commonalities but deviate on other aspects. For a more comprehensive discussion over the major differences, the reader is referred to Hjeij and Vilks (2023) and Samuels et al. (2012). The first school of thought is that proposed by Daniel Kahneman and Amos Tversky in the 1970s (Kahneman et al., 1974), who posit that heuristics are evolutionary mechanisms that use generalizations or rules-of-thumb to reduce cognitive load. They suggest that while they help us make quick decisions, they are often inaccurate. More importantly, we use heuristics even when there is little guarantee they will produce a correct answer. In short, Kahneman and Tversky suggest that human decision making is largely *irrational*.

The second school is that of bounded rationality started by Herbert Simon and continued by Gigerenzer (2015). One of the main contributions is the *adaptive toolbox* which defines heuristics as efficient processes which ignore information in favor of speed, but with one significant difference, that these heuristics are often ecologically *rational* and can provide answers that are nearly, or as-good as optimized decision-making mechanisms. Part of Gigerenzer’s toolbox was the concept of fast and frugal heuristics, where less information is more (Gigerenzer, 2008).

The difference between the two schools can be distilled into a difference in research focus. Kahneman and Tversky examined the *decisions* that people make (Kahneman & Klein, 2009), whereas Gigerenzer examines *cognitive mechanisms* (Gigerenzer & Gaissmaier, 2011). Both agree that if a heuristic is ill-fitted to the context, then they are biased and can be considered irrational. In our present research, we are examining the context of secure coding, for which no heuristic has evolved to handle (Oliveira et al., 2018), and so whether you subscribe primarily to Kahneman and Tversky’s definition of heuristics, or Gigerenzer’s, both approaches support the idea that in this context, intuitive, heuristic-based decisions are likely to produce incorrect and irrational decisions.

### Limitations

In this section, we draw attention to two domains of limitations: internal validity in terms of sampling and statistics, and external validity in terms of construct accuracy.

With respect to the former, we recognize that the work draws on self-reports of risk. We knew that asking participants about their experiences with developing software in the context of risk and uncertainty might prime security. All participants completed the same survey, and any priming effect would be across all participants. A different issue is that some participants responded to question prompts in a superficial or terse way. We asked respondents to write for at least 4 min or speak for 2 min per each qualitative, text-response question. Yet some responses were only three words long, limiting data quality. Typically, when this occurred, it was across all a participant’s text responses rather than specific questions. Drawing on a freelance online marketplace, we may have recruited participants who are focused on completing tasks quickly to maximize their hourly pay. Our preregistered sampling strategy was designed to mitigate data quality differences, proportions of language were used to ensure a relative association between response length and topic frequencies, and only responses longer than 20 words were analyzed. Nonetheless, further work that elicits richer data would be useful.

In the statistical (hurdle) model, the second step produced increased residuals in predicting high PURL, due to scarcer datapoints in the higher ranges. This could be a signal of a violation to the underlying distribution, however, Schielzeth et al. (2020) concluded mixed-effects models are relatively robust to such issues. We suggest this remains an appropriate analytic device, while welcoming any opportunity to complement these findings with convergent statistical approaches.

To measure optimism, we used a novel, software developer specific task, the OVT. The intention was to measure relative optimism between an individual and the “average” developer. Official statistics for the percentage of software applications suffering from specific OWASP vulnerabilities do not exist and so an absolute measure of optimism is unobtainable. By measuring relative optimism, we elicit information on domain-specific security concepts. The measure consists of two sections, one about personal risk and the other of the average developer. This was presented to participants in a fixed order which is like previous work using self-reported ratings of confidence (De Neys et al., 2013; Frederick, 2005; Hoover & Healy, 2019). Simplicity was valued and took research precedence in this regard, though of course it remains an open question as to whether different responses could be obtained through alternating section presentation. Note that a mixed order would have required additional model analytic terms, potentially resulting in overfitting, as well as reducing the analytic focus on the other cognitive measures.

Many studies have used CRT to understand individual differences in cognition. The first of two recurrent concerns is the extent to which performance is confounded by numerical ability, Although CRT associates strongly with numeracy (Liberali et al., 2012; Sinayev & Peters, 2015; Welsh et al., 2013), CRT responses have been shown to measure more than numeracy alone, as people tend to respond with a predictable intuitive and incorrect response (Pennycook & Ross, 2016). A second concern is interpretive; the attribution substitution hypothesis suggests that when using System 1 processing individuals unconsciously substitute complex decisions with computationally simpler ones (Hoover & Healy, 2019, 2021; Kahneman & Frederick, 2002). For our purposes, however, we simply note that showing the systematicity of the association between CRT scores and software cognition is the key first step. Disentangling the nuances of how conscious system switching or question framing might affect conceptual interpretation—of specific CRT questions—is left for more detailed enquiry.

### Future Work

Our future research will extend beyond the research described here, by leveraging preexisting paradigms (Brun et al., 2022) for measuring blindspots within application programming interfaces. By using a paradigm that reflects real-world scenarios, involving code reviews with insecure elements, measures of developers’ actions can be taken to associate with cognition. Previous work using this paradigm has not fully explored the relationships with heuristics and dual-processing theory which we anticipate being able to draw clear connections between. This will further evidence the need to examine software security through the lens of psychology to better understand the effect of cognition and individual differences in secure software development.

### Conclusion

Understanding how software engineers talk about risk and security in software is important as it provides insight into how they approach software development. The main finding from this study is that an interaction between cognitive reflection and optimism goes someway to explain risk sensitivity in language used by engineers when discussing software development, risk, and security. It was also seen that software developers and CS do not differ significantly in their approaches to security and risk. Additionally, software developers and CS both exhibit optimistic perceptions on their likelihood to include vulnerabilities in their own software. Future research should expand upon this work by performing similar measures alongside software development tasks.

## Supplementary Material

10.1037/tmb0000122.supp

## Figures and Tables

**Table 1 tbl1:** Reported Ages of Participants Included in the Analysis

Age	Developer	Student
18–24	28	42
25–34	34	20
35–44	6	8
45–54	1	2

**Table 2 tbl2:** Qualitative Questions Presented to Participants in the Order Listed

Number	Question
1	Describe a time when you successfully developed and released/launched a software project, either in a professional or personal capacity. This could either be a recent example, or perhaps a project you were particularly proud/happy with. Please include information concerning the purpose of the project and how important security was during development.
2	When considering the process of developing and launching software/web applications, what is at risk of potentially going wrong and how could these risks affect you? You should consider the size or the significance of the potential factors that may go wrong and how this may affect you (e.g., risk of functional failure, financial losses, damage to reputation, etc.)
3	If you were to consider software development as a series of ‘gambles’ (decisions that confer possible risk), what gambles would be considered worthwhile or worth a risk during the process of developing software? Why? These gambles may be considered from both an individual perspective and as a team. Both decisions that you take individually, or decisions that are enforced by policy, should be considered.
4	What approaches or considerations, do you, or your team, take when aiming to identify potential risks or security vulnerabilities when developing software? What is the reasoning behind these decisions? You should consider the decisions and thought processes behind selecting certain tools (such as static analysis tools), as well as identifying specific tools.
*Note*. Participants were asked to respond to these with either a written response or to record an audio response. Participants were requested to write for at least 4 min or speak for 2 min per question.	

**Table 3 tbl3:** The Raw Percentage Agreement Between the Primary Researcher and the Two Researchers Who Completed the Validation Task

Topic	Agreement
Risk	.87
Product	.88
Workplace	.86
Finance	.91
Geographic	.97
Legal	.91
Shallow skills	.94
*Note*. The two researchers both completed a separate 10% of the words available and so were combined allowing for the creation of Cohen’s κ between the primary researcher and the two researchers combined. Agreement is calculated through the confusion matrix and is the sum of both the primary and secondary researchers rating a word as true or false, divided by the sum total of words.	

**Table 4 tbl4:** Transformation of Optimism Task and PURL Scores Toward a Normal Distribution

Measure	Transform power	Shapiro–Wilks		*p* value	
		Original	Updated	Original	Updated
OVT	1.425	.96	.98	.006	.036
PURL	.05	.75	.99	<.001	.013
*Note*. Transform power is the optimal value identified through the Shapiro–Wilks test provided through using Tukey ladder of powers. The ladder applies a range of power transformations and reassesses the data for heteroscedasticity. The values listed under the Shapiro–Wilks section reflect the value before and after transformation, and the *p* value columns show the pre- and postvalues from the Shapiro–Wilks test. OVT = Open Web Application Security Project vulnerability task; PURL = proportion of uncertainty-related language.					

**Table 5 tbl5:** Model Coefficients of the Terms Used in the Modelling of the Presence of Uncertainty-Related Language

Term	β	Significance
Intercept	−1.35	***
CS prop	8.39	***
Question 2	1.91	***
Question 3	1.84	***
Question 4	0.96	***
Question 2: CSprop	−5.36	**
Question 3: CSprop	−2.6	
Question 4: CSprop	−0.35	
(1|participant)		
*Note*. The beta value reflects the model coefficient and is given alongside the significance of the term in the model. The terms relating to Questions 2, 3, and 4 are present through the inclusion of a categorical term of question, this is included as the questions ask about different ideas, and likely vary in frequency of PURL language. That is, the terms Questions 2, 3, and 4 represent the variability in the presence of these different questions. The intercept represents the first question, and so the question terms are interpreted in comparison to the first question, the model tells us that for Questions 2, 3, and 4, their beta values are positive compared to the first question indicating a higher likelihood of the presence of uncertainty-related language. PURL = proportion of uncertainty-related language; CSprop = proportion of Computer Science-related language.		
*p < .05. **p < .01. ***p < .001.		

**Table 6 tbl6:** Chosen Model for the Second Step Model Using Zero-Truncated PURL as a Dependent Variable

Term	β	Variance	Significance
Intercept	0.91		***
CRT	−0.04		**
OVT	−0.04		*
CRT × OVT	0.07		*
Question 2	0.01		***
Question 3	0.01		***
Question 4	0.02		***
Random (Question 1)		.0006	
Random (Question 2)		.0006	
Random (Question 3)		.0006	
Random (Question 4)		.0006	
*Note*. The terms listed are those present in the model for determining PURL in responses, with coefficient values listed in the beta column, along with confidence intervals in the CI column. The variance for the random effects is given providing a value for how much variation these terms provide and finally, the significance of each term for the model is given. The terms relating to the Questions 2, 3, and 4 are present through the inclusion of a categorical term of question, this is included as the questions ask about different ideas, and likely vary in frequency of PURL language. OVT = Open Web Application Security Project vulnerability task; PURL = proportion of uncertainty-related language; CRT = Cognitive Reflection Test; CI = confidence interval.			
*p < .05. **p < .01. ***p < .001.			

## Footnotes

1 The intuitive response is 10 cents, but the correct answer is 5 cents.

2 See section OWASP Vulnerability Task for further details.

3 Definitions can be found on the OWASP top ten list—https://owasp.org/www-project-top-ten/.

## Examples of Sentences and Their Transformed PURL Score

**Table:**

Subject ID	PURL	Sentence	Keywords
01	.00	I was assigned a project to handle a drinks factory day-to-day purchases, sales, and employee records three months ago.	N/A
89	.24	The main considerations I take when aiming to identify potential risks or security vulnerabilities when developing software are to consider the ways in which information could be compromised.	Risks, security, compromised
126	.35	Where personal experience is not available, I would regularly visit a risk assessment template throughout the development process and ensure that potential risks are regularly identified during the development lifecycle.	Risk, risks
09	.58	Good decisions always make software development smooth and secure, team lead would be a big factor, his ideas and decisions matters most	Decision, secure, decisions
143	.75	I would say that user data collected from already existing secured software will provide additional protection from risks, such as Gmail, bank accounts, and other similar places where there is already an existed security.	Secured, protection, risks, security
*Note*. PURL = proportion of uncertainty-related language; ID = identification number; N/A = not applicable.			

## References

[ref1] Abdellaoui M., Bleichrodt H., & Paraschiv C. (2007). Loss aversion under prospect theory: A parameter-free measurement. Management Science, 53(10), 1659–1674. 10.1287/mnsc.1070.0711

[ref2] Acar Y., Fahl S., & Mazurek M. L. (2016). You are not your developer, either: A research agenda for usable security and privacy research beyond end users [Conference session]. IEEE Cybersecurity Development, Boston, MA, United States. https://main.sec.uni-hannover.de/publications/conf-secdev-acarfm16/

[ref3] Acar Y., Stransky C., Wermke D., Weir C., Mazurek M. L., & Fahl S. (2017). Developers need support, too: A survey of security advice for software developers [Conference session]. 2017 IEEE Cybersecurity Development (SecDev), Cambridge, MA, United States. 10.1109/SecDev.2017.17

[ref4] Ajila C., & Abiola A. (2004). Influence of rewards on workers performance in an organization. Journal of Social Sciences, 8(1), 7–12. 10.1080/09718923.2004.11892397

[ref5] Alter A. L., & Oppenheimer D. M. (2008). Effects of fluency on psychological distance and mental construal (or why New York is a large city, but New York is a civilized jungle). Psychological Science, 19(2), 161–167. 10.1111/j.1467-9280.2008.02062.x 18271864

[ref6] Alter A. L., Oppenheimer D. M., Epley N., & Eyre R. N. (2007). Overcoming intuition: Metacognitive difficulty activates analytic reasoning. Journal of Experimental Psychology: General, 136(4), 569–576. 10.1037/0096-3445.136.4.569 17999571

[ref7] Assal H., & Chiasson S. (2018). Motivations and amotivations for software security [Conference session]. Fourteenth symposium on usable privacy and security (Vol. 4).

[ref8] Barreto C. F., & França C. (2021). Gamification in software engineering: A literature review [Conference session]. 2021 IEEE/ACM 13th international workshop on cooperative and human aspects of software engineering (CHASE), Madrid, Spain. 10.1109/CHASE52884.2021.00020

[ref9] Beike D. R., & Sherman S. J. (1994). Social inference; inductions, deductions, and analogies. In Wyer R. S. & Srull T. K. (Eds.), Handbook of social cognition (2nd ed., pp. 209–285) Lawrence Erlbaum.

[ref10] Białek M., & Pennycook G. (2018). The cognitive reflection test is robust to multiple exposures. Behavior Research Methods, 50(5), 1953–1959. 10.3758/s13428-017-0963-x 28849403

[ref11] Brun Y., Lin T., Somerville J. E., Myers E. M., & Ebner N. C. (2022). Blindspots in Python and Java APIs result in vulnerable code. ACM Transactions on Software Engineering and Methodology, 32(3), 1–31. 10.1145/3571850

[ref12] Cappos J., Zhuang Y., Oliveira D. S., Rosenthal M., & Yeh K.-C. (2014). Vulnerabilities as blind spots in developer’s heuristic-based decision-making processes [Conference session]. 2014 workshop on new security paradigms workshop—NSPW ‘14, Victoria, British Columbia, Canada. 10.1145/2683467.2683472

[ref13] Capretz L. F., & Ahmed F. (2018). A call to promote soft skills in software engineering. Psychology and Cognitive Sciences—Open Journal, 4(1), e1–e3. 10.17140/PCSOJ-4-e011

[ref14] Čavojová V., & Jurkovič M. (2017). Comparison of experienced vs. novice teachers in cognitive reflection and rationality. Studia Psychologica, 59(2), 100–112. 10.21909/sp.2017.02.733

[ref15] Chattopadhyay S., Nelson N., Au A., Morales N., Sanchez C., Pandita R., & Sarma A. (2020). A tale from the trenches: Cognitive biases and software development [Conference session]. ACM/IEEE 42nd international conference on software engineering, Seoul, South Korea. 10.1145/3377811.3380330

[ref16] Cohen J. (1960). A coefficient of agreement for nominal scales. Educational and Psychological Measurement, 20(1), 37–46. 10.1177/001316446002000104

[ref17] Damnjanović K., Novković V., Pavlović I., Ilić S., & Pantelić S. (2019). A cue for rational reasoning: Introducing a reference point in cognitive reflection tasks. Europe’s Journal of Psychology, 15(1), 25–40. 10.5964/ejop.v15i1.1701 PMC6396699 30915171

[ref18] Danilova A., Naiakshina A., Rasgauski A., & Smith M. (2021). Code reviewing as methodology for online security studies with developers—A case study with freelancers on password storage [Conference session]. Seventeenth USENIX Conference on Usable Privacy and Security, Berkeley, CA, United States.

[ref19] Dash N., & Gladwin H. (2007). Evacuation decision making and behavioral responses: Individual and household. Natural Hazards Review, 8(3), 69–77. 10.1061/(ASCE)1527-6988(2007)8:3(69)

[ref20] De Neys W., Rossi S., & Houdé O. (2013). Bats, balls, and substitution sensitivity: Cognitive misers are no happy fools. Psychonomic Bulletin & Review, 20(2), 269–273. 10.3758/s13423-013-0384-5 23417270

[ref21] Evans J. S. B. T. (1984). Heuristic and analytic processes in reasoning. British Journal of Psychology, 75(4), 451–468. 10.1111/j.2044-8295.1984.tb01915.x

[ref22] Evans J. S. B. T. (2003). In two minds: Dual-process accounts of reasoning. Trends in Cognitive Sciences, 7(10), 454–459. 10.1016/j.tics.2003.08.012 14550493

[ref23] Evans J. S. B. T. (2010a). Thinking twice: Two minds in one brain. Oxford University Press.

[ref24] Evans J. S. B. T. (2010b). Intuition and reasoning: A dual-process perspective. Psychological Inquiry, 21(4), 313–326. 10.1080/1047840X.2010.521057

[ref25] Evans J. S. B. T., & Stanovich K. E. (2013). Dual-process theories of higher cognition: Advancing the debate. Perspectives on Psychological Science, 8(3), 223–241. 10.1177/1745691612460685 26172965

[ref26] Fagerholm F., Felderer M., Fucci D., Unterkalmsteiner M., Marculescu B., Martini M., Tengberg L. G. W., Feldt R., Lehtelä B., Nagyváradi B., & Khattak J. (2022). Cognition in software engineering: A taxonomy and survey of a half-century of research. ACM Computing Surveys, 54(Suppl. 11), 1–36. 10.1145/3508359

[ref27] Frederick S. (2005). Cognitive reflection and decision making. Journal of Economic Perspectives, 19(4), 25–42. 10.1257/089533005775196732

[ref28] Gigerenzer G. (2002). Adaptive thinking: Rationality in the real world. Oxford University Press.

[ref29] Gigerenzer G. (2008). Why heuristics work. Perspectives on Psychological Science, 3(1), 20–29. 10.1111/j.1745-6916.2008.00058.x 26158666

[ref30] Gigerenzer G. (2015). Simply rational: Decision making in the real world. Oxford University Press.

[ref31] Gigerenzer G., & Gaissmaier W. (2011). Heuristic decision making. Annual Review of Psychology, 62(1), 451–482. 10.1146/annurev-psych-120709-145346 21126183

[ref32] Gigerenzer G., & Todd P. M. (1999). Simple heuristics that make us smart. Oxford University Press. 10.1017/s0140525x00003447 11301545

[ref33] Gotterbarn D. (2001). Informatics and professional responsibility. Science and Engineering Ethics, 7(2), 221–230. 10.1007/s11948-001-0043-5 11349362

[ref34] Hagoort P. (2023). The language marker hypothesis. Cognition, 230, Article 105252. 10.1016/j.cognition.2022.105252 36115201

[ref35] Hallett J., Patnaik N., Shreeve B., & Rashid A. (2021). “Do this! Do that!, And nothing will happen” Do specifications lead to securely stored passwords? [Conference session]. 43rd international conference on software engineering (ICSE ‘21), Madrid, ES, Spain. 10.1109/ICSE43902.2021.00053

[ref36] Hamari J. (2017). Do badges increase user activity? A field experiment on the effects of gamification. Computers in Human Behavior, 71, 469–478. 10.1016/j.chb.2015.03.036

[ref37] Hjeij M., & Vilks A. (2023). A brief history of heuristics: How did research on heuristics evolve? Humanities and Social Sciences Communications, 10(1), 1–15. 10.1057/s41599-023-01542-z

[ref38] Hoffmann A. O. I., Post T., & Pennings J. M. E. (2015). How investor perceptions drive actual trading and risk-taking behavior. Journal of Behavioral Finance, 16(1), 94–103. 10.1080/15427560.2015.1000332

[ref39] Hoover J. D., & Healy A. F. (2019). The bat-and-ball problem: Stronger evidence in support of a conscious error process. Decision, 6, 369–380. 10.1037/dec0000107 31632998 PMC6800670

[ref40] Hoover J. D., & Healy A. F. (2021). The bat-and-ball problem: A word-problem debiasing approach. Thinking & Reasoning, 27(4), 567–598. 10.1080/13546783.2021.1878473

[ref41] Ivory M. (2022). The soft skills of software learning development: The psychological dimensions of computing and security behaviours [Conference session]. International conference on evaluation and assessment in software engineering 2022, Gothenburg, Sweden. 10.1145/3530019.3535344

[ref42] Ivory M., Sturdee M., Towse J. N., Levine M., & Nuseibeh B. (2023). Can you hear the ROAR of software security? How Responsibility, Optimism And Risk shape developers’ security perceptions. PsyArXiv. 10.31234/osf.io/pexvz

[ref43] Ivory M., Towse J. N., Sturdee M., Levine M., & Nuseibeh B. (2022). Cognitive reflection, optimism and uncertainty in software development. 10.17605/OSF.IO/SJ8BT

[ref44] Jackendoff R. S. (2009). Language, consciousness, culture: Essays on mental structure. MIT Press.

[ref45] Janssen E. M., Meulendijks W., Mainhard T., Verkoeijen P. P. J. L., Heijltjes A. E. G., van Peppen L. M., & van Gog T. (2019). Identifying characteristics associated with higher education teachers’ Cognitive Reflection Test performance and their attitudes towards teaching critical thinking. Teaching and Teacher Education, 84, 139–149. 10.1016/j.tate.2019.05.008

[ref46] Jones H. S., Towse J., Race N., & Harrison T. (2019). Email fraud: The search for psychological predictors of susceptibility. PLOS ONE, 14(1), Article e0209684. 10.1371/journal.pone.0209684 30650114 PMC6334892

[ref47] Kahneman D., & Frederick S. (2002). Representativeness revisited: Attribute substitution in intuitive judgment. In Gilovich T., Griffin D., & Kahneman D. (Eds.), Heuristics and biases (1st ed., pp. 49–81). Cambridge University Press. 10.1017/CBO9780511808098.004

[ref48] Kahneman D., & Klein G. (2009). Conditions for intuitive expertise: A failure to disagree. American Psychologist, 64, 515–526. 10.1037/a0016755 19739881

[ref49] Kahneman D., Slovic P., & Tversky A. (Eds.). (1974). Judgment under uncertainty: Heuristics and biases (1st ed.). Cambridge University Press. 10.1126/science.185.4157.1124 17835457

[ref50] Kahneman D., & Tversky A. (1979). Prospect theory: An analysis of decision under risk. Econometrica, 47(2), 263–291. 10.2307/1914185

[ref51] Keil M., Wallace L., Turk D., Dixon-Randall G., & Nulden U. (2000). An investigation of risk perception and risk propensity on the decision to continue a software development project. The Journal of Systems and Software, 53, 145–157. 10.1016/S0164-1212(00)00010-8

[ref52] Kina K., Tsunoda M., Hata H., Tamada H., & Igaki H. (2016). Analyzing the decision criteria of software developers based on prospect theory [Conference session]. 2016 IEEE 23rd international conference on software analysis, evolution, and reengineering (SANER), Osaka, Japan. 10.1109/SANER.2016.115

[ref53] Kirlappos I., Beautement A., & Sasse M. A. (2013). “Comply or die” is dead: Long live security-aware principal agents. In Adams A. A., Brenner M., & Smith M. (Eds.), Financial cryptography and data security (pp. 70–82). Springer. 10.1007/978-3-642-41320-9_5

[ref54] Kula R. G., German D. M., Ouni A., Ishio T., & Inoue K. (2018). Do developers update their library dependencies?: An empirical study on the impact of security advisories on library migration. Empirical Software Engineering, 23(1), 384–417. 10.1007/s10664-017-9521-5

[ref55] Kunda Z. (1990). The case for motivated reasoning. Psychological Bulletin, 108, 480–498. 10.1037/0033-2909.108.3.480 2270237

[ref56] Levy J. S. (1992). An introduction to prospect theory. Political Psychology, 13(2), 171–186.

[ref57] Liberali J. M., Reyna V. F., Furlan S., Stein L. M., & Pardo S. T. (2012). Individual differences in numeracy and cognitive reflection, with implications for biases and fallacies in probability judgment. Journal of Behavioral Decision Making, 25(4), 361–381. 10.1002/bdm.752 23878413 PMC3716015

[ref58] Lopez T., Sharp H., Tun T., Bandara A., Levine M., & Nuseibeh B. (2019). Talking about security with professional developers [Conference session]. 2019 IEEE/ACM joint 7th international workshop on conducting empirical studies in industry (CESI) and 6th international workshop on software engineering research and industrial practice (SER IP), Montreal, QC, Canada. 10.1109/CESSER-IP.2019.00014

[ref59] Lopez T., Sharp H., Tun T., Bandara A. K., Levine M., & Nuseibeh B. (2022). Security responses in software development. ACM Transactions on Software Engineering and Methodology, 32(3), 1–29. 10.1145/3563211

[ref60] Lopez T., Tun T., Bandara A., Mark L., Nuseibeh B., & Sharp H. (2019). An anatomy of security conversations in stack overflow [Conference session]. 2019 IEEE/ACM 41st international conference on software engineering: Software engineering in society (ICSE-SEIS), Montreal, QC, Canada. 10.1109/ICSE-SEIS.2019.00012

[ref61] Loske A., Widjaja T., & Buxmann P. (2013, December 18). Cloud computing providers’ unrealistic optimism regarding IT security risks: A threat to users? ICIS 2013 proceedings. Association for Information Systems. https://aisel.aisnet.org/icis2013/proceedings/BreakthroughIdeas/11

[ref62] Manzoor F., Wei L., & Asif M. (2021). Intrinsic rewards and employee’s performance with the mediating mechanism of employee’s motivation. Frontiers in Psychology, 12, Article 563070. 10.3389/fpsyg.2021.563070 34335346 PMC8319625

[ref63] McAuliffe M., & Triandafyllidou A. (2021). World migration report 2022. International Organisation For Migration. https://publications.iom.int/books/world-migration-report-2022

[ref64] Mohanani R., Salman I., Turhan B., Rodríguez P., & Ralph P. (2020). Cognitive Biases in Software Engineering: A Systematic Mapping Study. IEEE Transactions on Software Engineering, 46(12), 1318–1339. 10.1109/TSE.2018.2877759

[ref65] Mølokken K., & Jørgensen M. (2005). Expert estimation of web-development projects: Are software professionals in technical roles more optimistic than those in non-technical roles? Empirical Software Engineering, 10(1), 7–30. 10.1023/B:EMSE.0000048321.46871.2e

[ref66] Moritz B., Siemsen E., & Kremer M. (2014). Judgmental Forecasting: Cognitive Reflection and Decision Speed. Production and Operations Management, 23(7), 1146–1160. 10.1111/poms.12105

[ref67] Nadi S., Krüger S., Mezini M., & Bodden E. (2016). Jumping through hoops: Why do Java developers struggle with cryptography APIs? [Conference session]. 38th international conference on software engineering, Austin, Texas. 10.1145/2884781.2884790

[ref68] Naiakshina A., Danilova A., Gerlitz E., von Zezschwitz E., & Smith M. (2019). “If you want, I can store the encrypted password”: A password-storage field study with freelance developers [Conference session]. 2019 CHI conference on human factors in computing systems, Glasgow, Scotland. 10.1145/3290605.3300370

[ref69] Oliveira D. S., Lin T., Rahman M. S., Akefirad R., Ellis D., Perez E., Bobhate R., DeLong L. A., Cappos J., & Brun Y. (2018). API blindspots: Why experienced developers write vulnerable code [Conference session]. Fourteenth USENIX Conference on Usable Privacy and Security (SOUPS ’18), Baltimore, MD, United States. 10.5555/3291228.3291253

[ref70] Oliveira D. S., Rosenthal M., Morin N., Yeh K.-C., Cappos J., & Zhuang Y. (2014). It’s the psychology stupid [Conference session]. 30th annual computer security applications conference, New Orleans, Louisiana, United States. 10.1145/2664243.2664254

[ref71] Palassis A., Speelman C. P., & Pooley J. A. (2021). An exploration of the psychological impact of hacking victimization. SAGE Open, 11(4). 10.1177/21582440211061556

[ref72] Palombo H., Tabari A. Z., Lende D., Ligatti J., & Ou X. (2020). An ethnographic understanding of software (in)security and a co-creation model to improve secure software development [Conference session]. Sixteenth Symposium on Usable Privacy and Security (SOUPS 2020), Berkeley, CA, United States. 10.5555/3488905.3488917

[ref73] Pennycook G., McPhetres J., Zhang Y., Lu J. G., & Rand D. G. (2020). Fighting COVID-19 misinformation on social media: Experimental evidence for a scalable accuracy-nudge intervention. Psychological Science, 31(7), 770–780. 10.1177/0956797620939054 32603243 PMC7366427

[ref74] Pennycook G., & Ross R. M. (2016). Commentary: Cognitive reflection vs. calculation in decision making. Frontiers in Psychology, 7, Article 9. 10.3389/fpsyg.2016.00009 26834682 PMC4722428

[ref75] Petre M. (2022). Exploring cognitive bias “in the wild”: Technical perspective. Communications of the ACM, 65(4), Article 114. 10.1145/3517215

[ref76] Ralph P. (2011). Toward a theory of debiasing software development. In Wrycza S. (Ed.), Research in systems analysis and design: Models and methods (pp. 92–105). Springer. 10.1007/978-3-642-25676-9_8

[ref77] Rauf I., Petre M., Tun T., Lopez T., Lunn P., Van Der Linden D., Towse J., Sharp H., Levine M., Rashid A., & Nuseibeh B. (2021). The case for adaptive security interventions. ACM Transactions on Software Engineering and Methodology, 31(1), 1–52. 10.1145/3471930

[ref78] Rhee H.-S., Ryu Y. U., & Kim C.-T. (2012). Unrealistic optimism on information security management. Computers & Security, 31(2), 221–232. 10.1016/j.cose.2011.12.001

[ref79] Roesler D. (2020). A computer science academic vocabulary list [Master’s thesis].

[ref80] Samuels R., Stich S., & Bishop M. (2012). Ending the rationality wars; How to make disputes about human rationality disappear. In Stich S. (Ed.), Collected papers: Vol. 2. Knowledge, rationality, and morality, 1978–2010 (pp. 236–268). Oxford University Press.

[ref81] Schielzeth H., Dingemanse N. J., Nakagawa S., Westneat D. F., Allegue H., Teplitsky C., Réale D., Dochtermann N. A., Garamszegi L. Z., & Araya-Ajoy Y. G. (2020). Robustness of linear mixed-effects models to violations of distributional assumptions. Methods in Ecology and Evolution, 11(9), 1141–1152. 10.1111/2041-210X.13434

[ref82] Sharot T. (2011). The optimism bias. Current Biology, 21(23), R941–R945. 10.1016/j.cub.2011.10.030 22153158

[ref83] Shreeve B., Gralha C., Rashid A., Araujo J., & Goulão M. (2022). Making sense of the unknown: How managers make cyber security decisions. ACM Transactions on Software Engineering and Methodology, 32(4), 1–33. 10.1145/3548682

[ref84] Siegrist M., Gutscher H., & Earle T. C. (2005). Perception of risk: The influence of general trust, and general confidence. Journal of Risk Research, 8(2), 145–156. 10.1080/1366987032000105315

[ref85] Sinayev A., & Peters E. (2015). Cognitive reflection vs. calculation in decision making. Frontiers in Psychology, 6, Article 532. 10.3389/fpsyg.2015.00532 25999877 PMC4423343

[ref86] Spadini D., Çalikli G., & Bacchelli A. (2020). Primers or reminders? The effects of existing review comments on code review [Conference session]. 2020 IEEE/ACM 42nd international conference on software engineering (ICSE), Seoul, South Korea.

[ref87] Stagnaro M., Pennycook G., & Rand D. G. (2018). Performance on the Cognitive Reflection Test is stable across time. Judgment and Decision Making, 13, 260–267. 10.1017/S1930297500007695

[ref88] Tahaei M., & Vaniea K. (2019). A survey on developer-centred security [Conference session]. 2019 IEEE European symposium on security and privacy workshops (EuroS&PW), Stockholm, Sweden. 10.1109/EuroSPW.2019.00021

[ref89] Tetlock P. E., & Kim J. I. (1987). Accountability and judgment processes in a personality prediction task. Journal of Personality and Social Psychology, 52, 700–709. 10.1037/0022-3514.52.4.700 3572733

[ref90] Thomson K. S., & Oppenheimer D. M. (2016). Investigating an alternate form of the cognitive reflection test. Judgment and Decision Making, 11(1), 99–113. 10.1017/S1930297500007622

[ref91] van der Linden D., Anthonysamy P., Nuseibeh B., Tun T., Petre M., Levine M., Towse J., & Rashid A. (2020). Schrödinger’s security: Opening the box on app developers’ security rationale [Conference session]. ACM/IEEE 42nd International Conference on Software Engineering, Seoul, South Korea. 10.1145/3377811.3380394

[ref92] Veracode. (2020). State of software security (Vol. 11). https://www.veracode.com/sites/default/files/pdf/resources/sossreports/state-of-software-security-volume-11-veracode-report.pdf

[ref93] von Eye A., & von Eye M. (2008). On the marginal dependency of Cohen’s K. European Psychologist, 13(4), 305–315. 10.1027/1016-9040.13.4.305

[ref94] Warrens M. J. (2014). On marginal dependencies of the 2 × 2 kappa. Advances in Statistics, 2014, Article e759527. 10.1155/2014/759527

[ref95] Weinstein N. D. (1980). Unrealistic optimism about future life events. Journal of Personality and Social Psychology, 39(5), 806–820. 10.1037/0022-3514.39.5.806

[ref96] Welsh M. B., Burns N. R., & Delfabbro P. H. (2013). The Cognitive Reflection Test: How much more than numerical ability? [Conference session]. Cognitive science conference (Vol. 7).

[ref97] West R. F. (2008). The psychology of security. The Psychology of Security, 51(4), 34–40. 10.1145/1330311.1330320

[ref98] Wiederhold B. K. (2014). The role of psychology in enhancing cybersecurity. Cyberpsychology, Behavior, and Social Networking, 17(3), 131–132. 10.1089/cyber.2014.1502 24592869

